# Effects of solid‐phase extraction of plasma in measuring gut metabolic hormones in fasted and fed blood of lean and diet‐induced obese rats

**DOI:** 10.14814/phy2.12800

**Published:** 2016-05-20

**Authors:** Roger Reidelberger, Alvin Haver, Krista Anders, Bettye Apenteng, Craig Lanio

**Affiliations:** ^1^ Department of Biomedical SciencesCreighton UniversityOmahaNebraska; ^2^ VA Research ServiceVA Nebraska Western Iowa Health Care SystemOmahaNebraska; ^3^ EMD Millipore Corp.BillericaMassachusetts

**Keywords:** Amylin, ghrelin, GLP‐1, intravenous infusion, PYY(3‐36)

## Abstract

Glucagon‐like peptide‐1 (GLP‐1), peptide YY (3‐36) [PYY(3‐36)], amylin, ghrelin, insulin, and leptin are thought to act as hormonal signals from periphery to brain to control food intake. Here, we determined the effects of solid‐phase extraction of plasma in measuring these hormones in blood of lean and diet‐induced obese rats. Individual enzyme‐linked immunoassays and a multiplex assay were used to measure active GLP‐1, total PYY, active amylin, active ghrelin, insulin, leptin, and total GIP in response to (1) addition of known amounts of the peptides to lean and obese plasma, (2) a large meal in lean and obese rats, and (3) intravenous infusions of anorexigenic doses of GLP‐1, PYY(3‐36), amylin, and leptin in lean rats. Extraction of lean and obese plasma prior to assays produced consistent recoveries across assays for GLP‐1, PYY, amylin, ghrelin, and insulin, reflecting losses inherent to the extraction procedure. Plasma extraction prior to assays generally revealed larger meal‐induced changes in plasma GLP‐1, PYY, amylin, ghrelin, and insulin in lean and obese rats. Plasma extraction and the multiplex assay were used to compare plasma levels of GLP‐1, PYY, and amylin after a large meal with plasma levels produced by IV infusions of anorexigenic doses of GLP‐1, PYY(3‐36), and amylin. Infusions produced dose‐dependent increases in plasma peptide levels, which were well above their postprandial levels. These results do not support the hypothesis that postprandial plasma levels of GLP‐1, PYY(3‐36), and amylin are sufficient to decrease food intake by an endocrine mechanism.

## Introduction

The gut metabolic hormones – glucagon‐like peptide‐1 (GLP‐1), peptide YY (3‐36) [PYY(3‐36)], and amylin – are thought to act as hormonal signals from gut to brain to produce satiety (reduce food intake). Increase in food intake in response to peripheral administration of their receptor antagonists suggest that these peptides play essential roles in producing satiety (Reidelberger et al. 2004, 2013; Williams et al. 2009).

Although several studies suggest that GLP‐1 and PYY(3‐36) may act through paracrine stimulation of intestinal vagal sensory neurons to reduce food intake, others suggest that anorexic responses to GLP‐1, PYY(3‐36), and amylin may be mediated in part or entirely by nonvagal pathways (Halatchev and Cone 2005; Talsania et al. 2005; Rüttimann et al. 2009; Zhang and Ritter 2012; Mietlicki‐Baase and Hayes 2014; Reidelberger et al. 2014). If these peptides act as blood‐borne signals, it would be important to determine whether food intake is inhibited by intravenous (IV) doses that reproduce meal‐induced increases in their plasma levels. If inhibition were to occur for a peptide, this would suggest that the postprandial increase in the peptide is sufficient to inhibit food intake by an endocrine mechanism. If little or no effect was observed, then either the peptide does not inhibit food intake by an endocrine mechanism or the circulating peptide interacts with other factor(s) in an additive or potentiating manner to produce satiety.

Several human studies suggest that postprandial increases in plasma GLP‐1 are sufficient to decrease food intake (Verdich et al. 2001). In rats, IV infusion of GLP‐1 at 33 ng/kg/min increases plasma GLP‐1 to a level comparable to that produced by food intake (Tolessa et al. 1998), and we have shown that this dose is sufficient to decrease food intake (Chelikani et al. 2005b). In contrast, other studies have shown little or no change in plasma GLP‐1 in response to food intake in humans and rats (Brennan et al. 2005; Dai et al. 2008; Shin et al. 2010; Yabe et al. 2012, 2015; Bak et al. 2014; Punjabi et al. 2014). With respect to PYY(3‐36), postprandial plasma levels do not appear to be sufficient to inhibit food intake (Beglinger and Degen 2006; Stadlbauer et al. 2013). With respect to amylin, we have provided evidence that postprandial levels in rats are not quite sufficient to inhibit food intake (Arnelo et al. 1998). However, a wide variability in basal and stimulated concentrations of plasma GLP‐1, PYY(3‐36), and amylin has been reported by different investigators, which likely reflects differences in antisera and standards used in individual assays, and whether plasma samples were extracted to remove interfering factors before being assayed (Dai et al. 2008; Deacon and Holst 2009; Yabe et al. 2012).

Here, we determined the effects of solid‐phase extraction of plasma in measuring GLP‐1, PYY(3‐36), amylin, ghrelin, insulin, leptin, and GIP in blood of lean and diet‐induced obese (DIO) rats. Individual enzyme‐linked immunoassays (ELIAs) and a multiplex assay were used to measure active GLP‐1, total PYY, active amylin, active ghrelin, insulin, leptin, and total GIP in response to (1) addition of known amounts of the various peptides to lean and obese plasma, (2) a large meal in lean and obese rats, and (3) IV infusions of anorexigenic doses of GLP‐1, PYY(3‐36), amylin, and leptin in lean rats. We then compared the postprandial plasma levels of GLP‐1, PYY, and amylin with those produced by their anorexigenic doses.

## Materials and Methods

### GLP‐1, PYY(3–36), amylin, and leptin

Amylin was purchased from Bachem Americas, Inc. (Torrance, CA). Rat PYY(3–36) and GLP‐1 were synthesized by Fmoc solid‐phase methodology (Amblard et al. 2006) and purified by reverse‐phase high‐performance liquid chromatography. Proof of structure was provided by electrospray mass spectrometry. Recombinant mouse leptin was obtained from Dr. A. F. Parlow, National Hormones and Peptides Program, Harbor‐UCLA Medical Center, Los Angeles, CA. Leptin was dissolved at 50 nmol/mL in phosphate‐buffered saline at pH 8. One‐milliliter aliquots were stored at −70°C until the day of use.

### Animals

The Animal Studies Subcommittee of the Omaha Veterans Affairs Medical Center approved the protocol. Outbred male lean and DIO rats (CD IGS; Charles River, Inc., Kingston, NY) were used in experiments. There appears to be no consensus in defining obesity in rats, although percent body fat above 20% body weight is likely (Novelli et al. 2007). Here, we induced obesity (≥25% body fat) as described previously (Reidelberger et al. 2011) with a 45% fat diet (D12451, 4.73 kcal/g; Research Diets, Inc., New Brunswick, NJ) and water ad libitum over a 4–6 month period. Total body fat mass was determined noninvasively using quantitative magnetic resonance (QMR, EchoMRI 700; Echo Medical Systems, Houston, TX). Lean rats were provided rat chow (Labdiet, 5001 Rodent diet, 3.3 kcal/g; PMI Nutrition International, Brentwood, MO) and water ad libitum.

### Jugular vein catheterization

Procedures for implanting a jugular vein catheter for administering GLP‐1, PYY(3‐36), amylin, and leptin were described previously (Woltman et al. 1995). Catheters were filled with heparinized saline (40 U/mL), plugged with stainless steel wire, and flushed every other day to maintain patency. The animals were allowed at least 1 week to recover from surgery. Rats were then adapted for at least 1 week to being chronically tethered to infusion swivels. The catheter was connected to a 40 cm long tubing passing through a protective spring coil connected between a light‐weight harness worn by the rat and an infusion swivel (Instech Laboratories, Inc., Plymouth Meeting, PA). Except where noted below, rats had free access to ground chow that was provided fresh each day 3 h before dark onset.

### Effects of food intake on plasma levels of active GLP‐1, total PYY, active amylin, active ghrelin, insulin, leptin, and total GIP in lean, and DIO rats

Our objective was to determine the effects of solid‐phase extraction of plasma, using two different commercial assay systems (an ELIA for each hormone and a multiplex assay), in measuring the various metabolic hormones in the same samples of fasted and fed blood. To accomplish this, each blood sample had to be relatively large, which limited the number of samples that could be obtained and analyzed from the rats. Thus, we chose only two sampling times – one after an overnight fast in one group of rats, and another 30 min after meal onset in a second group of rats because numerous studies have shown that meals in humans produce near‐maximal increases in plasma GLP‐1, PYY, and amylin within 30 min of meal onset, which are sustained for several hours (Arnelo et al. 1998; Verdich et al. 2001; Vilsbøll et al. 2001, 2003; Batterham et al. 2003, 2006; Ludvik et al. 2003; Degen et al. 2005; English et al. 2006; Yabe et al. 2010; Beglinger et al. 2014; Meyer‐Gerspach et al. 2014; Alsalim et al. 2015; Østoft et al. 2015). Rats were adapted for several days to an overnight fast, which causes them to immediately eat ~4 times the amount of food (2/3 of total daily intake) that they would normally eat during the first hour after returning food (Kinzig et al. 2005). We observed but did not measure the conditioned, hyperphagic response for each rat. Blood was sampled by cardiac puncture in anesthetized rats (a terminal procedure) because relatively large samples (2–4 mL) could be quickly obtained. Each blood sample provided the required 1 mL of plasma for extraction, which provided 0.45 mL of reconstituted extract for the various assays (described further in *Solid‐phase extraction of plasma samples*). This volume was not sufficient to perform all assays because sample volumes required for the multiplex assay, and the ELIAs for GLP‐1, PYY, amylin, ghrelin, insulin, leptin and GIP, were 0.05, 0.2, 0.1, 0.1 0.04, 0.02, 0.02, and 0.02 mL, respectively, totaling 0.55 mL. Thus, we conducted three identical experiments using different groups of rats to determine the effects of plasma extraction and food intake on plasma levels of the various hormones. Experiment 1 measured GLP‐1 in lean rats using the multiplex assay and ELIA. Experiment 2 measured PYY, amylin, ghrelin, insulin, leptin, and GIP in lean rats using the ELIAs, and these peptides as well as GLP‐1 using the multiplex assay. Experiment 3 measured GLP‐1, PYY, amylin, ghrelin, insulin, leptin, and GIP in DIO rats using only the multiplex assay because (1) results from Experiments 1 and 2 showed that in lean rats, plasma peptide responses to food intake were generally similar regardless of assay employed if plasma samples were extracted before assay, and (2) we did not have a sufficient number of DIO rats to perform the experiment twice in order to generate the volume of reconstituted extract needed to perform all ELIAs as well as the multiplex assay.

In each experiment, rats were provided food (chow for lean rats; 45% fat diet for obese rats) from 900 to 1400 h each day for several days before blood collection. Rats were then either provided food or further deprived of food for 30 min at 900 h, followed by anesthetization with isoflurane (3% to effect) and sampling of blood (~2–4 mL) by cardiac puncture. Samples were immediately transferred to 4 mL EDTA tubes on ice containing 40 *μ*L of a mixture of enzyme inhibitors: 0.9 mL DPP4 inhibitor (DPP4; EMD Millipore, St. Charles, MO), 0.1 mL Protease Inhibitor Cocktail (P2714; Sigma‐Aldrich, St. Louis, MO), and 0.1 g AEBSF (Pefabloc SC, Sigma‐Aldrich). Plasma was obtained by centrifugation within 30 min of sample collection and stored at −70°C.

### Effects of IV infusions of anorexigenic doses of GLP‐1, PYY(3‐36), amylin, and leptin on plasma levels of active GLP‐1, total PYY, active amylin, and leptin

We previously determined the dose‐response effects of 3‐h IV infusions of GLP‐1, PYY(3‐36), amylin, and leptin in reducing food intake in lean rats (Reidelberger et al. 2001; Chelikani et al. 2005a,b). Numerous studies have shown that continuous IV infusions of GLP‐1, PYY(3‐36), and amylin for several hours increase their plasma concentrations to steady‐state levels within 30 min of infusion onset that are linearly related to peptide dose and sustained for the duration of infusions (Nauck et al. 1997; Flint et al. 1998; Gutzwiller et al. 1999b; Näslund et al. 1999; Brennan et al. 2005; Degen et al. 2005; Young 2005; Ito et al. 2006; Witte et al. 2009; Asmar et al. 2010; Plamboeck et al. 2015). Here, we measured the plasma levels of active GLP‐1, total PYY, active amylin, and leptin 45 min after onset of IV infusions of anorexigenic doses of the peptides in lean rats.

After an overnight fast, rats with jugular vein catheters maintained on a chow diet were divided into three weight‐matched groups of seven rats each [449 ± 23, 445 ± 22, and 490 ± 15 g; *F*(2, 18) = 1.5, *P* > 0.05]. The first group received a continuous IV infusion of a combination of GLP‐1, PYY(3‐36), amylin, and leptin at 56, 68, 39, and 480 ng/kg/min, respectively, the second group received these peptides at 165, 200, 118, and 1600 ng/kg/min, and the third group received vehicle (0.15 mol/L NaCl, 0.1% bovine serum albumin, 2 mL/h). The lower doses of GLP‐1, PYY(3‐36), and amylin were ~ED_50_ doses for reducing food intake in lean rats [76, 60, and 31 ng/kg/min, respectively (Reidelberger et al. 2001; Chelikani et al. 2005a,b)]. Leptin doses bracketed the minimal effective dose (1100 ng/kg/min) for reducing food intake in lean rats (Reidelberger et al. 2012). Approximately 45 min after infusion onset, and while the infusion continued, rats were anesthetized with isoflurane (3%), and blood samples (~3 mL) were obtained by cardiac puncture. Samples were immediately transferred to 4 mL EDTA tubes on ice containing 40 *μ*L of a mixture of 0.9 mL DPP4 inhibitor, 0.1 mL Protease Inhibitor Cocktail, and 0.1 g AEBSF. Plasma was obtained by centrifugation within 30 min of sample collection and stored at −70°C until solid‐phase extraction of samples followed by the multiplex assay. Infusions were administered using a syringe infusion pump (Harvard Apparatus, South Natick, MA). At the end of the experiment, data from a rat were excluded if its jugular vein catheter was not patent. A catheter was deemed patent if the rat lost consciousness within 10 sec of a bolus injection of the short‐acting anesthetic brevital into the catheter.

#### Solid‐phase extraction of plasma samples

Plasma samples were extracted using procedures similar to those described previously (Dai et al. 2008). Briefly, an OASIS HLB LP (60 mg) 96‐well extraction plate (Waters Corp., Milford, MA) was prepared by passing 1 mL acetonitrile twice through each well, followed by 3, 1 mL washes of 1% trifluoroacetic acid (TFA; 1:100 water [vol/vol]). Flows through the wells were facilitated using a Waters vacuum manifold (5 inch Hg). Plasma (1 mL) was then added to each well; 10 min later, flows were facilitated using vacuum. Wells were then washed three times with 1 mL 1% TFA (facilitated using vacuum). Extracts were eluted into individual tubes by adding 1 mL of 1% TFA, 60% acetonitrile in water (vol/vol) to each well; 10 min later, flows were facilitated using vacuum. Samples of each extract (0.9 mL) were frozen, and then lyophilized to dryness. At time of assay, dried extracts for each sample were dissolved with 0.45 mL of assay buffer, producing a twofold higher concentration for eluted plasma components.

#### Peptide assays

Assays of active GLP‐1, total PYY, active amylin, active ghrelin, insulin, leptin and total GIP in unextracted and extracted plasma samples were performed using a multiplex magnetic bead‐based kit (Milliplex, RMHMAG‐84K; EMD Millipore) and individual ELIAs for GLP‐1 (EGLP‐35K), amylin (EZHA‐52K), ghrelin (EZRGA‐90K), insulin (EZRMI‐13K), leptin (EZRL‐83K) and GIP (EZRMGIP‐55K) (all from EMD Millipore), and a ELIA for PYY (EK‐059‐03; Phoenix Pharmaceuticals, Inc., Burlingame, CA). The multiplex assay employed a BioTek ELx405 plate washer fitted with a magnetic holder, and a Luminex 200 analyzer. Intra‐ and inter‐assay coefficients of variation ranged from 1–3% and 7–13%, respectively. Initial assays compared peptide standards used in the multiplex assay with those used in the individual ELIAs: multiplex assay standards were included in the individual ELIAs, and standards from the ELIAs were included in the multiplex assay.

The next series of multiplex assays and ELIAs determined the effects of solid‐phase extraction of plasma samples on specific recoveries of the seven peptides (multiplex standards) added at three different concentrations to pools of assay buffer and plasma collected from fasted lean and fed DIO rats. We chose plasma from fed DIO rats to assess whether extraction recoveries are influenced by high blood lipid, which is common when DIO rats consume the 45% fat diet. Expected increases in plasma peptide concentrations were 120, 600, and 3000 pg/mL for GLP‐1 and amylin; 20, 100, and 500 pg/mL for PYY and ghrelin; 200, 1000, and 5000 pg/mL for insulin and leptin; and 8, 40, and 200 pg/mL for GIP. Two different recovery experiments were performed. The first used both the multiplex assay and the individual ELIAs to measure peptide concentrations in unextracted and extracted lean plasma. Blood was collected from donor rats into EDTA tubes, and plasma was obtained by centrifugation and stored at −70°C. Enzyme inhibitors (10 *μ*L of a mixture of 0.9 mL DPP4 inhibitor, 0.1 mL protease inhibitor cocktail, and 0.1 g AEBSF per mL of plasma) were added after thawing and pooling of plasma, just prior to addition of peptides. Extractions of samples from the same plasma pools were performed in triplicate. The second experiment was of identical design, except the mixture of enzyme inhibitors was added to EDTA tubes at time of blood collection from donor rats, and only the multiplex assay was used to measure peptide concentrations in extracted lean and obese plasma. For the multiplex assay and the insulin, ghrelin, GIP, and leptin ELIAs, matrix solution included in the individual assay kits was added to a second set of assay standards according to manufacturer's instructions. Standards without matrix were used to assay extracted plasma samples and standards with matrix were used to assay unextracted plasma samples.

### Statistical analysis

In experiments examining the effects of solid‐phase extraction of plasma samples on specific recoveries of the seven peptides added to lean and obese plasma, measured peptide concentrations in each plasma sample were plotted against expected concentrations (after subtracting plasma alone values), and linear regression analyses were performed. The coefficient of determination (*R* ^2^) from each analysis provided a statistical measure of how well the data fit the regression line across the range of peptide amounts added to plasma. The slope of the regression line provided a measure of % recovery of peptide added to plasma. Slopes were compared using the method of Cohen (2003). Linear regression analysis was used to define relationships between dose of peptide infused IV and the plasma peptide level produced by the dose. Multifactorial ANOVA was used to determine the effects of food intake, ELIA versus multiplex assay, plasma extraction, and body composition (lean vs. obese) on plasma concentrations of active GLP‐1, total PYY, active amylin, active ghrelin, insulin, leptin, and total GIP. Planned comparisons of treatment means were evaluated by *t*‐tests or paired *t*‐tests. Differences were considered significant if *P < *0.05. Values are shown as group means ± SE, or group means followed by a 95% confidence interval within brackets.

## Results

### Peptide assays

Using the multiplex peptide standards as reference standards, the standards used in the individual ELIAs were 300% higher for GLP‐1, 10% lower for PYY, 33% lower for amylin, 50% lower for ghrelin, 37% lower for insulin, 11% higher for leptin, and 29% lower for GIP. In most of the analyses, data obtained using the individual ELIAs were adjusted accordingly to enable comparisons with multiplex assay data.

**Figure 1:**
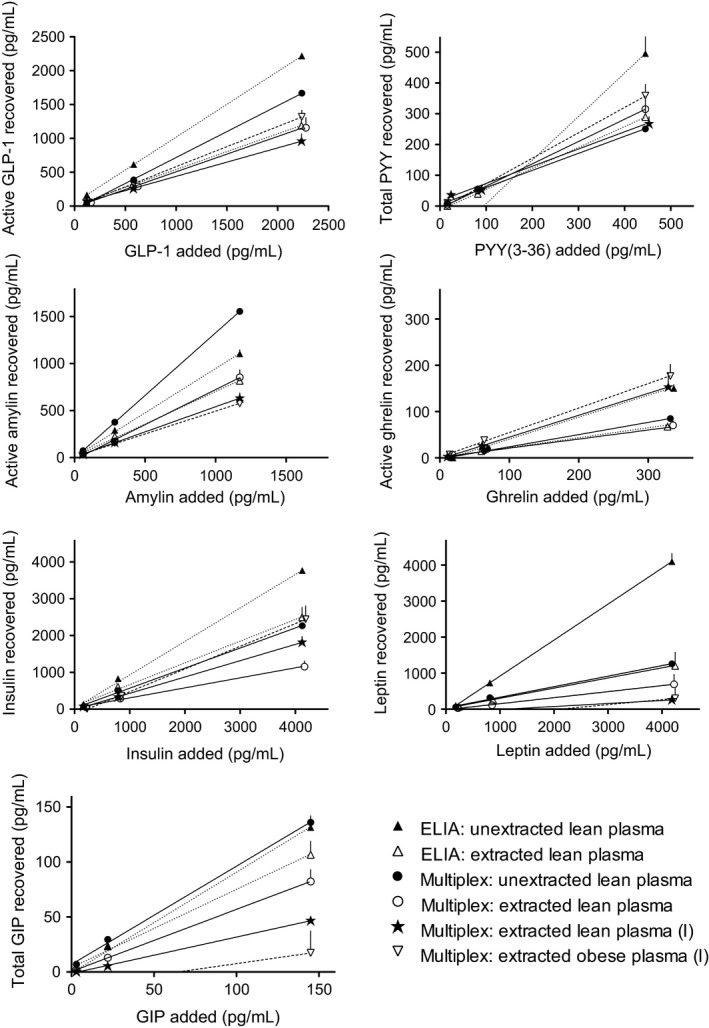
Effects of solid‐phase extraction of lean and obese plasma on assay specificity. Known amounts of peptides were added to pools of assay buffer, lean plasma, and obese plasma. Enzyme inhibitors were added at time of peptide addition to pools, or at time of blood collection (I). Individual ELIAs and the multiplex assay were used to measure peptide levels in samples of assay buffer, plasma, and plasma extracts. Multiplex standards were used as reference standards. Lines depict linear regression results. Values are group means ± SE.

Mixtures of the multiplex standards containing GLP‐1, PYY(3‐36), amylin, ghrelin, insulin, leptin, and GIP were added at three different concentrations to pools of assay buffer, lean plasma, and obese plasma. Percent recoveries of these peptides before and after solid‐phase extraction of plasma samples were determined using both the individual ELIAs and the multiplex assay. Average peptide concentrations in assay buffer were 138, 734, and 2946 pg/mL for GLP‐1; 19, 84, and 381 pg/mL for PYY; 97, 452, and 2296 pg/mL for amylin; 17, 79, and 393 pg/mL for ghrelin; 179, 911, and 4430 pg/mL for insulin; 256, 942, and 4733 pg/mL for leptin; and 6, 30 and 168 pg/mL for GIP. Figure 1 shows assay measurements of the peptides in lean and obese plasma before and after extraction of plasma samples, plotted against expected concentrations of the peptides in the plasma samples. Table 1 provides the linear regression slopes and their *R* ^2^ goodness of fit. Slopes of regression lines (times 100) provided estimates of percentage recoveries for each peptide under each condition. When unextracted samples from spiked lean plasma were assayed using the individual ELIAs, mean peptide recoveries were between 90 and 100% for all peptides except PYY and ghrelin, which were 142% and 47%, respectively. For comparison, when unextracted samples from the same spiked plasma pools were assayed using the multiplex assay, average recoveries were lower for GLP‐1, PYY, ghrelin, insulin, and leptin (77%, 55%, 26%, 55%, and 29%, respectively, all *P* < 0.001 except *P* < 0.01 for PYY), larger for amylin (133%, *P* < 0.001), and similar for GIP (89%, *P* > 0.05). When 1 mL samples from the same pools of lean plasma were extracted before assay, the individual ELIAs and the multiplex assay gave similar recoveries for GLP‐1, PYY, amylin, and GIP (ranging from 52% to 75%, all *P* > 0.05), while insulin recovery was in the same range for the ELIA (59%, *P* > 0.05) and significantly lower for the multiplex assay (27%, *P* < 0.001). When using the ELIA to measure leptin, plasma extraction significantly reduced recovery of leptin (28 vs. 100% with no extraction, *P* < 0.0001), and when using the multiplex assay to measure leptin, extraction did not improve upon the low leptin recovery observed with no extraction (17 vs. 29% with no extraction, *P* > 0.05).

**Table 1:** Effects of solid‐phase extraction of lean and DIO plasma on assay specificity

	ELIA unextracted lean		Multiplex unextracted lean		ELIA extracted lean		Multiplex extracted lean		Multiplex extracted lean^a^		Multiplex extracted obese^a^	
	Slope	*R* ^2^	Slope	*R* ^2^	Slope	*R* ^2^	Slope	*R* ^2^	Slope	*R* ^2^	Slope	*R* ^2^
GLP‐1	0.97 ± 0.01	1.00	0.77 ± 0.01^b^	1.00	0.53 ± 0.03	0.98	0.52 ± 0.05	0.94	0.42 ± 0.06	0.84	0.59 ± 0.03^d^	0.98
PYY(3‐36)	1.42 ± 0.19	0.97	0.55 ± 0.01^b^	1.00	0.68 ± 0.06	0.94	0.72 ± 0.05	0.96	0.56 ± 0.06	0.90	0.83 ± 0.06^d^	0.96
Amylin	0.94 ± 0.03	1.00	1.33 ± 0.01^b^	1.00	0.69 ± 0.02	1.00	0.75 ± 0.05	0.97	0.53 ± 0.05	0.90	0.49 ± 0.04	0.96
Ghrelin	0.47 ± 0.02	0.99	0.26 ± 0.01^b^	1.00	0.22 ± 0.03	0.89	0.20 ± 0.02	0.93	0.47 ± 0.10	0.68	0.53 ± 0.06	0.92
Insulin	0.91 ± 0.01	1.00	0.55 ± 0.01^b^	1.00	0.59 ± 0.05	0.95	0.27 ± 0.03^c^	0.93	0.44 ± 0.04	0.91	0.62 ± 0.07	0.92
Leptin	1.00 ± 0.04	0.99	0.29 ± 0.01^b^	0.99	0.28 ± 0.08	0.69	0.17 ± 0.05	0.63	0.08 ± 0.02	0.53	0.14 ± 0.07	0.37
GIP	0.92 ± 0.04	0.99	0.89 ± 0.04	0.99	0.71 ± 0.06	0.95	0.56 ± 0.05	0.94	0.33 ± 0.03	0.91	0.21 ± 0.10	0.38

Regression statistics [slopes and coefficients of determination (*R* ^2^)] are for data presented in Figure 1.

a Inhibitors were added at time of blood collection.

b *P* < 0.05 versus ELIA unextracted lean.

c *P* < 0.05 versus ELIA extracted lean.

d *P* < 0.05 versus Multiplex extracted lean; values are means ± SE.

A second experiment used only the multiplex assay to measure peptide recoveries in extracted lean and obese plasma. Here, the enzyme inhibitors were added to EDTA tubes at the time of blood collection from donor rats rather than to pools of thawed plasma just prior to addition of peptides. Recoveries were larger for ghrelin and insulin, and smaller for GIP, whether plasma was from lean or obese rats (Table 1). When comparing peptide recoveries from lean and obese plasma, recoveries were similar for each peptide except GLP‐1 and PYY, which were slightly larger when added to obese plasma (GLP‐1: 59% vs. 42%, *P* < 0.05; PYY(3‐36): 83% vs. 56%, *P* < 0.01).

### Experiment 1: effects of food intake and plasma extraction on plasma levels of active GLP‐1 in lean rats

**Figure 2:**
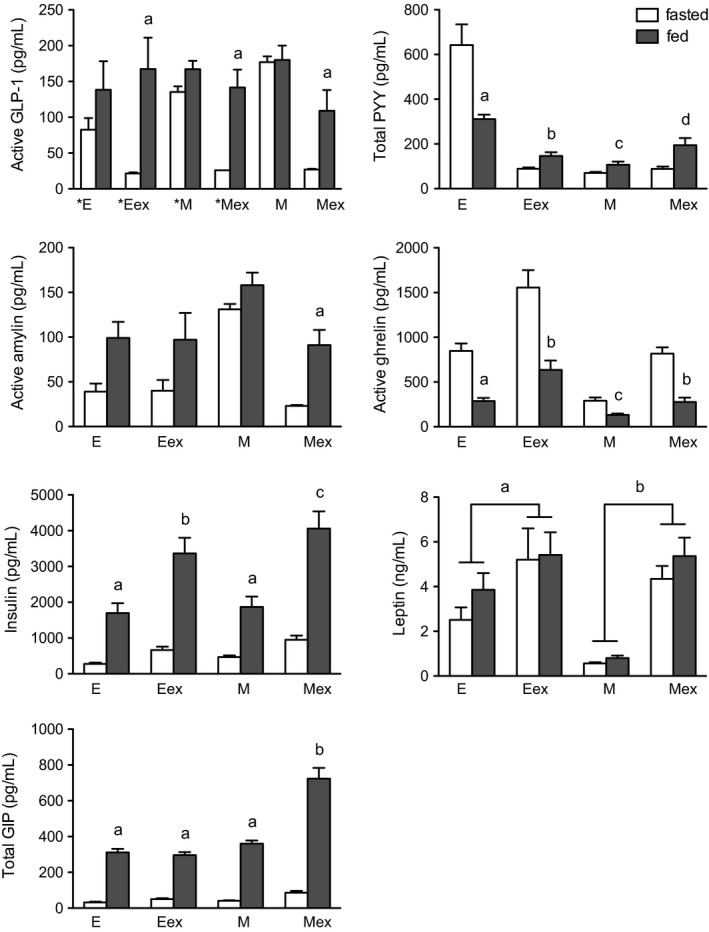
Effects of food intake and solid‐phase plasma extraction on plasma levels of active GLP‐1, total PYY, active amylin, active ghrelin, insulin, leptin, and total GIP in lean rats. Overnight fasted rats were offered chow; blood was collected at the end of fast or 30 min after meal onset. Individual ELIAs and the multiplex assay were used to measure the peptide levels in samples of plasma and plasma extracts. Multiplex standards were used as reference standards. E, ELIA; ex, extracted plasma; M, multiplex assay.*, Experiment 1 data. Treatment means with letters are significantly different when compared to mean fasting peptide levels. Feeding responses with different letters are significantly different (a, b, c, d: *P *<* *0.05), except as noted for leptin. Values are group means ± SE.

This experiment determined the effects of food intake on plasma GLP‐1 using the different assay methodologies (no plasma extraction vs. plasma extraction; ELIA vs. multiplex assay). Ten rats were adapted to a 19‐h overnight fast. Blood samples were taken from five rats (509 ± 15 g, 9 ± 1% fat mass) after the 19‐h fast, and from another five rats (508 ± 14 g, 11 ± 1% fat mass) 30 min after access to rat chow following the 19‐h fast. A 3‐factor ANOVA (food intake [fasted vs. fed, between subjects]; assay [ELIA vs. multiplex assay, within subjects]; and plasma extraction [unextracted vs. extracted, within subjects]) showed significant main effects of food intake [*F*(1, 7) = 10.9, *P* < 0.05] and extraction [*F*(1, 7) = 15.7, *P* < 0.01] on plasma GLP‐1, and significant interactions between food intake and extraction [*F*(1, 7) = 16.3, *P* < 0.01] and assay and extraction [*F*(1, 7) = 7.1, *P* < 0.05]. Thus, plasma extraction revealed a significant stimulatory effect of food intake on active GLP‐1 in plasma that was independent of assay employed (Fig. 2). When using the multiplex assay to measure GLP‐1 in extracted plasma, basal levels were below the detection limit of 26 pg/mL, and postprandial levels were 142 ± 25 pg/mL. When using the ELIA to measure GLP‐1 in the same plasma extracts, basal and postprandial levels were 7.3 ± 0.7 [5.9, 8.7] and 56 ± 15 [27, 85] pg/mL, respectively. Since concentrations of the GLP‐1 standards in the multiplex assay were 67% lower when measured in the GLP‐1 ELIA, the multiplex‐derived values for basal and postprandial levels are reduced to <8.6 and 46 ± 8.2 [30, 62] pg/mL, when ELIA standards are used as reference standards.

### Experiment 2: effects of food intake and plasma extraction on plasma levels of total PYY, active amylin, active ghrelin, insulin, leptin, and total GIP in lean rats

This experiment in a second group of lean rats determined the effects of food intake and plasma extraction on plasma levels of the other hormones (total PYY, active amylin, active ghrelin, insulin, leptin, total GIP) using the different assay methodologies. Fifteen rats were adapted to a 19‐h overnight fast. Blood samples were taken from eight rats (476 ± 3 g, 12 ± 3% fat mass) after the 19‐h fast, and from another seven rats (471 ± 3 g, 14 ± 3% fat mass) 30 min after access to rat chow following the 19‐h fast. Although active GLP‐1 was not measured here using its ELIA, active GLP‐1 was measured with the other peptides in the multiplex assay. Results showed the same effects of food intake and plasma extraction on GLP‐1 levels as observed in Experiment 1 when the multiplex assay was used to measure GLP‐1 (Fig. 2). A 3‐factor ANOVA showed significant main effects of food intake [*F*(1, 21) = 16.1, *P* < 0.001] and extraction [*F*(1, 21) = 105, *P* < 0.001] on GLP‐1 levels, and a significant interaction between food intake and extraction [*F*(1, 21) = 22.5, *P* < 0.001]; yet, there was no main effect of experiment [*F*(1, 21) = 0.2, *P* > 0.05] or interaction of experiment with food intake and extraction [*F*(1, 21) = 0.03, *P* > 0.05].

With respect to plasma total PYY, the 3‐factor ANOVA showed no main effect of food intake [*F*(1, 13) = 1.0, *P* > 0.05], yet significant interactions between food intake and extraction [*F*(1, 13) = 21.3, *P* < 0.001], and food intake, extraction, and assay [*F*(1, 13) = 9.5, *P* < 0.01]. Plasma extraction revealed a significant meal‐induced increase in total PYY, which appeared larger in the multiplex assay (Fig. 2). When using the multiplex assay to measure total PYY in extracted plasma, food intake increased PYY from 88 ± 11 to 194 ± 32 pg/mL. When using the ELIA to measure total PYY in the same plasma extracts, basal and postprandial plasma levels were 89 ± 6 [77, 100] and 146 ± 17 [110, 180] pg/mL, respectively. However, the multiplex standards were 10% higher in concentration when measured in the ELIA. Thus, the multiplex‐derived values for basal and postprandial levels are 96 ± 12 [72, 120] and 210 ± 35 [140, 280] pg/mL, respectively, when ELIA standards are used as reference standards. Food intake appeared to reduce plasma total PYY when the ELIA was used to measure PYY in unextracted plasma. However, the manufacturer's instructions for the ELIA recommended plasma extraction to remove high levels of interfering proteins that can affect assay results. Thus, our results suggest that fasting plasma samples may have contained higher levels of these factors than the postprandial samples, which came from different groups of rats.

With respect to plasma active amylin, the 3‐factor ANOVA showed no significant main effects or interactions of food intake, extraction, and assay. There were too few measureable results from the ELIA to compare extraction effects across assay methodologies (Fig. 2). Plasma extraction revealed a significant meal‐induced increase in plasma amylin, when the multiplex assay was employed (Fig. 2). Food intake increased plasma amylin from 23 ± 1 to 91 ± 17 pg/mL. However, the multiplex standards were 50% higher in concentration when measured in the ELIA. Thus, the multiplex‐derived values for basal and postprandial levels are 34 ± 1.6 [31, 37] and 140 ± 26 [89, 190] pg/mL, respectively, when ELIA standards are used as reference standards.

With respect to plasma active ghrelin, the 3‐factor ANOVA showed significant main effects of food intake [*F*(1, 12) = 38.3, *P* < 0.0001], extraction [*F*(1, 12) = 110, *P* < 0.0001], and assay [*F*(1, 12) = 198, *P* < 0.0001], and significant interactions for each combination of food intake, extraction, and assay [e.g., food intake, extraction, and assay: *F*(1, 12) = 8.4, *P* < 0.05]. Plasma ghrelin levels were generally higher when samples were assayed with the ELIA; food intake significantly decreased plasma ghrelin, and plasma extraction similarly enhanced the feeding response across assays (Fig. 2). When using the multiplex assay to measure active ghrelin in extracted plasma, food intake decreased plasma ghrelin from 820 ± 69 to 280 ± 48 pg/mL. When using the ELIA to measure active ghrelin in the same plasma extracts, basal and postprandial plasma levels were 1600 ± 200 [1210, 2000] and 640 ± 100 [440, 840] pg/mL, respectively. However, the multiplex standards were 200% higher in concentration when measured in the ELIA. Thus, the multiplex‐derived values for basal and postprandial levels are 1620 ± 140 [1350, 1900] and 560 ± 96 [370, 750] pg/mL, respectively, when ELIA standards are used as reference standards.

With respect to plasma insulin, the 3‐factor ANOVA showed significant main effects of food intake [*F*(1, 12) = 35.4, *P* < 0.001], extraction [*F*(1, 12) = 52.6, *P* < 0.0001], and assay [*F*(1, 12) = 200, *P* < 0.001], and significant interactions for each combination of food intake, extraction, and assay [e.g., food intake, extraction, and assay: *F*(1, 12) = 30.6, *P* < 0.001]. Food intake produced a significant increase in plasma insulin independent of extraction and assay, and plasma extraction enhanced the feeding response when the multiplex assay was employed (Fig. 2). When using the multiplex assay to measure insulin in extracted plasma, food intake increased plasma insulin from 950 ± 110 to 4100 ± 480 pg/mL. When using the ELIA to measure insulin in the same plasma extracts, basal and postprandial plasma levels were 670 ± 96 [480, 860] and 3400 ± 440 [2540, 4260] pg/mL, respectively. However, the multiplex standards were 60% higher in concentration when measured in the ELIA. Thus, the multiplex‐derived values for basal and postprandial levels are 1500 ± 190 [1130, 1870] and 6600 ± 780 [5070, 8130] pg/mL, respectively, when ELIA standards are used as reference standards.

With respect to plasma leptin, the 3‐factor ANOVA showed no main effect of food intake [*F*(1, 8) = 0.3, *P* > 0.05], significant main effects of extraction [*F*(1, 8) = 26.8, *P* < 0.001] and assay [*F*(1, 8) = 31.2, *P* < 0.001], and a significant interaction between extraction and assay [*F*(1, 13) = 17.6, *P* < 0.01]. Food intake did not change plasma leptin, yet leptin levels were significantly higher when measured using the ELIA, and were enhanced by extraction when using both assays (Fig. 2). However, as noted above, extraction recoveries were quite low with leptin in both assays. When using the multiplex assay to measure leptin in unextracted plasma, basal and postprandial plasma levels were 560 ± 52 and 800 ± 120 pg/mL, respectively. When using the ELIA to measure leptin in the same unextracted samples, basal and postprandial plasma levels were 2500 ± 560 [1400, 3600] and 3900 ± 740 [2450, 5350] pg/mL, respectively. However, the multiplex standards were 10% lower in concentration when measured in the ELIA. Thus, the multiplex‐derived values for basal and postprandial levels are 500 ± 49 [404, 596] and 730 ± 97 [540, 920] pg/mL, respectively, when ELIA standards are used as reference standards.

With respect to plasma total GIP, the 3‐factor ANOVA showed significant main effects of food intake [*F*(1, 11) = 263, *P* < 0.0001], extraction [*F*(1, 11) = 23.7, *P* < 0.001], and assay [*F*(1, 11) = 67.9, *P* < 0.001], and significant interactions for each combination of food intake, extraction, and assay [e.g., food intake, extraction, and assay: *F*(1, 11) = 39, *P* < 0.001]. Food intake produced a significant increase in plasma total GIP, and plasma extraction enhanced the feeding response only when the multiplex assay was employed (Fig. 2). When using the multiplex assay to measure total GIP in unextracted plasma, food intake increased plasma GIP from 41 ± 3 to 360 ± 18 pg/mL. When using the multiplex assay to measure total GIP in extracted plasma, food intake increased plasma GIP from 86 ± 10 to 720 ± 60 pg/mL. When using the ELIA to measure total GIP in the same unextracted samples, basal and postprandial levels were 32 ± 4 and 311 ± 20 pg/mL, respectively. When using the ELIA to measure total GIP in the same plasma samples after extraction, basal and postprandial levels were 50 ± 5 [40, 60] and 300 ± 17 [270, 330] pg/mL, respectively. Since multiplex standards were 40% higher in concentration when measured in the ELIA, multiplex‐derived values for basal and postprandial levels are 60 ± 5 and 500 ± 25 pg/mL, respectively, for unextracted plasma, and 120 ± 15 [91, 150] and 1000 ± 85 [830, 1170] pg/mL, respectively, for extracted plasma, when ELIA standards are used as reference standards.

### Experiment 3: effects of food intake and plasma extraction on plasma levels of active GLP‐1, total PYY, active amylin, active ghrelin, insulin, leptin, and total GIP in DIO rats

A similar experiment in DIO rats used only the multiplex assay to determine the effects of food intake and plasma extraction on plasma active GLP‐1, total PYY, active amylin, active ghrelin, insulin, leptin, and total GIP. Fifteen rats were adapted to a 19‐h overnight fast. Blood samples were taken from eight rats (850 ± 18 g, 36 ± 2% fat mass) after a 19‐h fast, and from another seven rats (812 ± 12 g, 31 ± 1% fat mass) 30 min after access to food following the 19‐h fast. Results were compared with those described for lean rats in Experiments 1 and 2 when using the multiplex assay to determine the effects of food intake and plasma extraction on plasma peptide levels.

**Figure 3:**
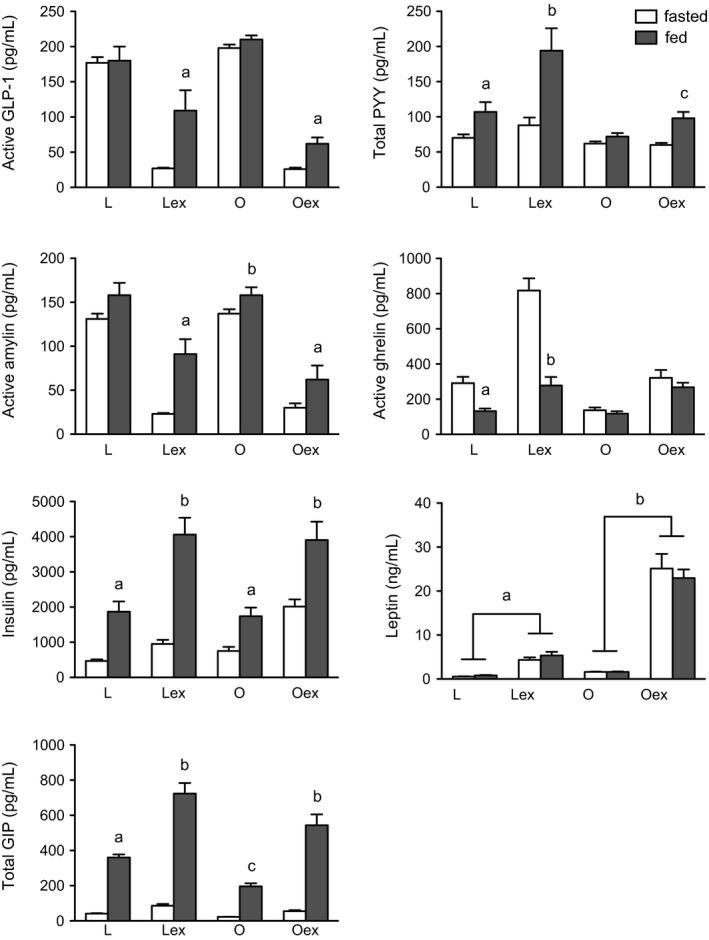
Effects of food intake and solid‐phase plasma extraction on plasma levels of active GLP‐1, total PYY, active amylin, active ghrelin, insulin, leptin, and total GIP in lean and DIO rats. Overnight fasted rats were offered chow or high‐fat food; blood was collected at the end of fast or 30 min after meal onset. The multiplex assay was used to measure the peptide levels in samples of plasma and plasma extracts. Multiplex standards were used as reference standards. L, lean plasma; ex, extracted plasma; O, obese plasma. Treatment means with letters are significantly different when compared to mean fasting peptide levels. Feeding responses with different letters are significantly different (a, b, c, d: *P *<* *0.05), except as noted for leptin. Values are group means ± SE.

With respect to plasma active GLP‐1, the 3‐factor ANOVA (food intake [fasted vs. fed, between subjects], body composition [lean vs. obese, between subjects], and plasma extraction [unextracted vs. extracted, within subjects]) showed significant main effects of food intake [*F*(1, 26) = 10.1, *P* < 0.01] and extraction [*F*(1, 26) = 347, *P* < 0.001], and significant interactions between food intake and extraction [*F*(1, 26) = 13.1, *P* < 0.01] and obesity and extraction [*F*(1, 26) = 11.6, *P* < 0.01]. Food intake increased plasma active GLP‐1, and plasma extraction enhanced the feeding response independently of body composition [interaction among food intake, body composition, and extraction: *F*(1, 26) = 3.6, *P* > 0.05] (Fig. 3).

With respect to plasma total PYY, the 3‐factor ANOVA showed significant main effects of food intake [*F*(1, 26) = 17.6, *P* < 0.001], body composition [*F*(1, 26) = 13.6, *P* < 0.001], and extraction [*F*(1, 26) = 31, *P* < 0.001], and significant interactions between food intake and body composition [*F*(1, 26) = 4.5, *P* < 0.05], food intake and extraction [*F*(1, 26) = 17.3, *P* < 0.001], and body composition and extraction [*F*(1, 26) = 12.1, *P* < 0.01]. Food intake increased plasma total PYY, more in lean than DIO rats, and plasma extraction enhanced the feeding response more in lean rats (Fig. 3).

With respect to plasma active amylin, the 3‐factor ANOVA showed significant main effects of food intake [*F*(1, 26) = 17.1, *P* < 0.001] and extraction [*F*(1, 26) = 417, *P* < 0.001], and a significant interaction between food intake and extraction [*F*(1, 26) = 8.1, *P* < 0.01]. Food intake increased plasma active amylin, and plasma extraction enhanced the feeding response independently of body composition [interaction among food intake, body composition, and extraction: *F*(1, 26) = 2.5, *P* > 0.05] (Fig. 3).

With respect to plasma active ghrelin, the 3‐factor ANOVA showed significant main effects of food intake [*F*(1, 26) = 31.9, *P* < 0.001], body composition [*F*(1, 26) = 31.9, *P* < 0.001], and extraction [*F*(1, 26) = 159, *P* < 0.001], and significant interactions for all combinations of food intake, body composition, and extraction [e.g., food intake, body composition, and extraction: *F*(1, 26) = 18.9, *P* < 0.001]. Food intake decreased plasma active ghrelin in lean but not DIO rats, and plasma extraction enhanced this feeding response (Fig. 3).

With respect to plasma insulin, the 3‐factor ANOVA showed significant main effects of food intake [*F*(1, 26) = 48.9, *P* < 0.001] and extraction [*F*(1, 26) = 199, *P* < 0.001], and a significant interaction of food intake and extraction [*F*(1, 26) = 36.7, *P* < 0.001]. Food intake increased plasma insulin independently of body composition, and plasma extraction enhanced this feeding response (Fig. 3).

With respect to plasma leptin, the 3‐factor ANOVA showed significant main effects of body composition [*F*(1, 26) = 90, *P* < 0.001] and extraction [*F*(1, 26) = 167, *P* < 0.001], and a significant interaction of body composition and extraction [*F*(1, 26) = 78.5, *P* < 0.001]. Food intake had no effect on plasma leptin, leptin was increased in DIO rats, and plasma extraction increased leptin levels, more so in DIO rats (Fig. 3). However, as noted above, extraction recoveries were quite low with leptin in both assays.

With respect to plasma total GIP, the 3‐factor ANOVA showed significant main effects of food intake [*F*(1, 26) = 312, *P* < 0.001], body composition [*F*(1, 26) = 18.4, *P* < 0.001], and extraction [*F*(1, 26) = 107, *P* < 0.001], and significant interactions of food intake and body composition [*F*(1, 26) = 10.3, *P* < 0.01], and food intake and extraction [*F*(1, 26) = 69, *P* < 0.001]. Food intake increased plasma total GIP more in lean than DIO rats, and plasma extraction enhanced feeding responses independently of body composition (Fig. 3).

### Effects of IV infusions of anorexigenic doses of GLP‐1, PYY(3‐36), amylin, and leptin on plasma levels of active GLP‐1, total PYY, active amylin, and leptin in lean rats

**Figure 4:**
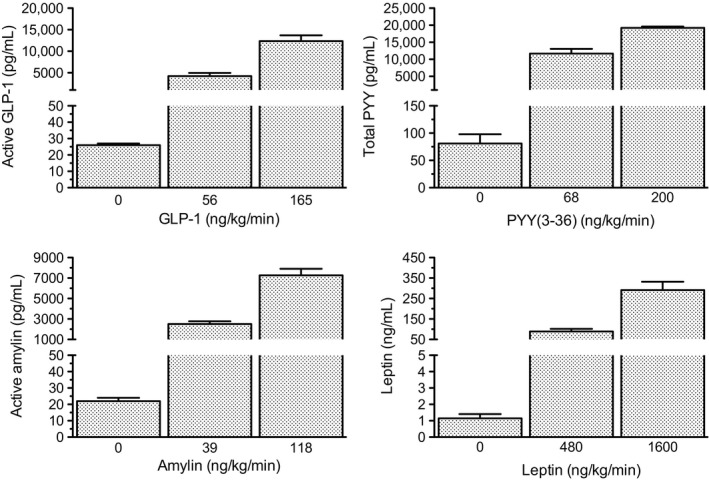
Effects of IV infusions of anorexigenic doses of GLP‐1, PYY(3‐36), amylin, and leptin on plasma levels of active GLP‐1, total PYY, active amylin, and leptin in lean rats. Overnight fasted rats received continuous IV infusions of vehicle or a combination of these peptides at two different doses each. Approximately 45 min after infusion onset, rats were anesthetized and blood was collected by cardiac puncture. The multiplex assay was used to measure hormone levels in plasma extracts. Multiplex standards were used as reference standards. Values are group means ± SE.

IV infusions of different doses of GLP‐1 (0, 56, 165 ng/kg/min), PYY(3‐36) (0, 68, 200 ng/kg/min), amylin (0, 39, 118 ng/kg/min), and leptin (0, 480, 1600 ng/kg/min) for 45 min in unanesthetized lean rats produced dose‐dependent increases in plasma active GLP‐1, total PYY, active amylin, and leptin, when measured in extracted plasma samples using the multiplex assay and multiplex standards as reference standards (Fig. 4). Best‐fit linear regression equations were: Y = 37 + 75X for GLP‐1 [*R* ^2^ = 0.87, *F*(1, 18) = 117, *P* < 0.001], Y = 2217 + 92X for PYY [*R* ^2^ = 0.85, *F*(1, 18) = 100, *P* < 0.001], Y = 60 + 61X for amylin [*R* ^2^ = 0.92, *F*(1, 18) = 197, *P* < 0.001], and Y = 1686 + 181X for leptin [*R* ^2^ = 0.83, *F*(1, 18) = 89, *P* < 0.001], where X is peptide dose (ng/kg/min) and Y is plasma peptide concentration (pg/mL). When using ELIA standards as reference standards, the ~ED_50_ doses of GLP‐1, PYY(3‐36), and amylin administered here (56, 68, and 39 ng/kg/min, respectively) produced plasma peptide levels (1400 [920, 1890], 12,800 [9870, 15,820], and 3760 [2960, 4560] pg/mL, respectively) that were 30–60 fold higher than those produced by the large meal (46 [30, 62], 210 [140, 280], and 140 [89, 190] pg/mL, respectively). Regression analysis was used to predict plasma peptide levels produced by previously reported minimal anorexigenic doses of GLP‐1, PYY(3‐36), and amylin [1.6, 20, and 3.9 ng/kg/min, respectively (Reidelberger et al. 2001; Chelikani et al. 2005a,b)], which were not administered here. Predicted levels were 53 [−400, 510], 4400 [2200, 6600], and 450 [−460, 1360] pg/mL for active GLP‐1, total PYY, and active amylin, respectively.

## Discussion

We used individual ELIAs and a multiplex assay to determine the effects of solid‐phase extraction of plasma in measuring active GLP‐1, total PYY, active amylin, active ghrelin, insulin, leptin, and total GIP in blood of lean and DIO rats. Known amounts of GLP‐1, PYY(3‐36), amylin, ghrelin, insulin, leptin, and GIP were added to plasma, and extraction recoveries were determined for each peptide. Results were as follows: (1) When enzyme inhibitors (DPP4 inhibitor, protease inhibitor cocktail, AEBSF) and the seven peptides were added to lean plasma pools just prior to performing the individual ELIAs on pool samples, recoveries were 90–100% for GLP‐1, amylin, insulin, leptin, and GIP, 142% for PYY, and 47% for ghrelin. (2) When samples from the same plasma pools were assayed using the multiplex assay, peptide recoveries were more variable, ranging from 26% to 133%. (3) When samples from the same plasma pools were extracted prior to assay, the ELIAs and multiplex assay showed similar recoveries for GLP‐1, PYY, amylin, and GIP (ranging from 52% to 75%), different recoveries for insulin (59% and 27%, respectively), and low recoveries for ghrelin (22% and 20%, respectively) and leptin (28% and 17%, respectively). (4) When the enzyme inhibitors were immediately added to blood at time of collection from lean rats [recommended for measuring active GLP‐1 (Kuhre et al. 2015)], and samples of spiked plasma pools were extracted and then assayed using only the multiplex assay, recoveries for GLP‐1, PYY, amylin, ghrelin, and insulin ranged from 42% to 56%, while those for leptin and GIP were only 8% and 33%, respectively. (5) When samples of spiked obese plasma pools were assayed under the same conditions, extraction recoveries were similar to those observed for lean plasma except for GLP‐1 and PYY, which were slightly higher (59% and 83%, respectively). Thus, extraction of the lean and obese plasma samples prior to assays produced consistent recoveries across assays for GLP‐1, PYY, amylin, ghrelin, and insulin, reflecting losses inherent to the extraction procedure.

Numerous studies have shown that meals in humans produce near‐maximal increases in plasma GLP‐1, PYY, and amylin within 30 min of meal onset, which are sustained for several hours (Arnelo et al. 1998; Verdich et al. 2001; Vilsbøll et al. 2001, 2003; Batterham et al. 2003, 2006; Ludvik et al. 2003; Degen et al. 2005; English et al. 2006; Yabe et al. 2010; Beglinger et al. 2014; Meyer‐Gerspach et al. 2014; Alsalim et al. 2015; Østoft et al. 2015). Here, we used the individual ELIAs and multiplex assay to determine the effects of solid‐phase extraction of plasma samples in measuring plasma levels of active GLP‐1, total PYY, active amylin, active ghrelin, insulin, leptin and total GIP before and 30 min after onset of a large meal in lean and DIO rats after an overnight fast. Results showed the following: (1) In lean rats, food intake increased plasma GLP‐1, PYY, amylin, insulin, and GIP, decreased ghrelin, and had no effect on leptin. Plasma extraction revealed and/or enhanced feeding responses for GLP‐1, PYY, amylin (multiplex only), ghrelin, insulin, and GIP (multiplex only), with the ELIAs and multiplex assay showing similar responses for GLP‐1, PYY, amylin, and insulin. (2) In DIO rats, plasma extraction revealed smaller meal‐induced responses for PYY, ghrelin, and GIP, and a trend for smaller GLP‐1 and amylin responses, when compared to those observed in lean rats. It remains to be determined whether these differences were due in part to differences in composition and/or size of meals ingested by the lean and DIO rats. (3) Food intake did not change leptin levels in either lean or DIO rats, although leptin levels were higher in DIO rats. Together, these results indicate that when using the assays employed in this study, solid‐phase extraction of rat plasma using the OASIS system revealed larger meal‐induced changes in plasma levels of active GLP‐1, total PYY, active amylin, active ghrelin, and insulin.

Shin et al. (2010) employed the same or similar multiplex and GLP‐1 ELIA assays to measure changes in plasma levels of active GLP‐1, total PYY, active amylin, active ghrelin, insulin, leptin, and total GIP in lean rats in response to a relatively small liquid meal (Ensure, 5 kcal) after an overnight fast. The same protease inhibitors were added to blood samples at time of collection to prevent degradation of peptides, but plasma samples were not extracted prior to assay. When their results were compared to those observed here when plasma samples were also not extracted, fasting plasma levels were similar for total PYY, active ghrelin, insulin, and total GIP, higher for leptin, and lower for active amylin and active GLP‐1 (not detectable), while peak meal‐induced changes in plasma levels were similar for active GLP‐1 (no change), total PYY, active ghrelin, and total GIP, and larger for active amylin and insulin. In contrast, their meal‐induced responses were smaller when compared to those observed here when plasma samples were extracted before assay. It remains to be determined whether these differences were due in part to differences in rat strain or composition and amount of food consumed.

We previously determined the dose–response effects of 3‐h IV infusions of GLP‐1, PYY(3‐36), and amylin in reducing food intake in lean rats: ED_50_ doses were 76, 60, and 31 ng/kg/min, respectively, and minimal effective doses were 1.6, 20, and 3.9 ng/kg/min, respectively (Reidelberger et al. 2001; Chelikani et al. 2005a,b). Numerous studies have shown that continuous IV infusions of GLP‐1, PYY(3‐36), and amylin for several hours increase their plasma concentrations to steady‐state levels within 30 min of infusion onset that are linearly related to peptide dose and sustained for the duration of infusions (Nauck et al. 1997; Flint et al. 1998; Gutzwiller et al. 1999b; Näslund et al. 1999; Brennan et al. 2005; Degen et al. 2005; Young 2005; Ito et al. 2006; Witte et al. 2009; Asmar et al. 2010; Plamboeck et al. 2015). Here, we measured the plasma levels of active GLP‐1, total PYY, and active amylin in lean rats 45 min after onset of IV infusions of anorexigenic doses of GLP‐1, PYY(3‐36), and amylin (~ED_50_ and threefold higher doses). Infusions produced dose‐dependent increases in plasma levels of each peptide. The ~ED_50_ doses produced plasma levels of GLP‐1, PYY, and amylin (1400 [920, 1890], 12,800 [9870, 15,820], and 3760 [2960, 4560] pg/mL, respectively) that were 30–60 fold higher than those produced by the large meal (46 [30, 62], 210 [140, 280], and 140 [89, 190] pg/mL, respectively). Regression analysis was used to predict plasma peptide levels produced by the minimal anorexigenic doses of GLP‐1, PYY(3‐36), and amylin [1.6, 20, and 3.9 ng/kg/min, respectively (Reidelberger et al. 2001; Chelikani et al. 2005a,b)], which were not administered here. Predicted plasma concentrations were 53 [−400, 510], 4400 [2200, 6600], and 450 [−460, 1360] pg/mL, respectively. Although the confidence intervals for GLP‐1 and amylin overlap with those for postprandial increases in plasma GLP‐1 and amylin, their large sizes preclude detecting practical differences between meal‐induced and infusion‐induced plasma peptide levels, if they truly existed (Type II error). Together, these results do not support the hypothesis that postprandial plasma levels of GLP‐1, PYY, and amylin are sufficient to decrease food intake by an endocrine mechanism.

Previous studies using two different ELIAs to measure meal‐induced changes in active GLP‐1 in human plasma suggest that accurate measurement of GLP‐1 requires addition of DPP4 inhibitor to blood samples at time of collection to prevent degradation of active GLP‐1, use of an assay specific for active GLP‐1, and either ethanol or solid‐phase extraction of plasma samples to remove nonspecific interference in the assays (Dai et al. 2008; Yabe et al. 2012; Kuhre et al. 2015). Our results here in rats confirm this contention for the active GLP‐1 ELIA, and extend it to the multiplex assay for active GLP‐1. Previous studies using rats and the same GLP‐1 ELIA showed little or no meal‐induced increase in GLP‐1 in systemic blood; however, only relatively small meals were consumed and plasma samples were not extracted (Shin et al. 2010; Punjabi et al. 2014).

Human studies employing DPP4 inhibitor, plasma extraction, and an ELIA specific for active GLP‐1 showed either no increase (Yabe et al. 2010, 2015) or relatively small increases in postprandial plasma levels of active GLP‐1 [from 2.6 to 9.9 pg/mL (Dai et al. 2008), 3.3 to 8.2 pg/mL (Meyer‐Gerspach et al. 2014), and 6.6 to 23 pg/mL (Østoft et al. 2015)]. In contrast, Gibbons et al. (2013) reported larger postprandial increases in active GLP‐1 (from 20 to 53 pg/mL) in overweight/obese humans when using DPP4 inhibitor, a multiplex assay, and no plasma extraction. It remains to be determined whether these larger values were due in part to differences in body weight of subjects or concentrations of standards used in the ELIA and multiplex assays, and whether plasma extraction would reveal an even larger meal‐induced response.

If endogenous GLP‐1 acts by an endocrine mechanism to produce satiety, it would be important to determine whether food intake is inhibited by IV doses of GLP‐1 that reproduce meal‐induced increases in plasma GLP‐1. Although numerous studies have addressed this issue using different GLP‐1 assays, none have provided conclusive evidence because either DPP4 inhibitor was not added to blood samples at time of collection (Flint et al. 1998, 2001; Gutzwiller et al. 1999a,b; Näslund et al. 1999; Brennan et al. 2005), plasma samples were not extracted before assay (Brennan et al. 2005; Shin et al. 2010; Punjabi et al. 2014), or a specific assay for active GLP‐1 was not employed (Flint et al. 1998, 2001; Gutzwiller et al. 1999a,b; Brennan et al. 2005; Plamboeck et al. 2015). Here, we used a DDP4 inhibitor, plasma extraction, and specific assays for active GLP‐1 to show that a large chow meal increased plasma GLP‐1 from below the detection limit of 8.6 to 46 ± 8.2 pg/mL, and that a ~ED_50_ dose of GLP‐1 produced 30‐fold higher levels. It remains to be determined whether IV infusion of GLP‐1 in a manner that mimics this postprandial increase, is sufficient to reduce food intake.

With respect to the gut hormone PYY(3‐36), previous studies suggest that postprandial plasma levels of PYY(3‐36) are not sufficient to inhibit food intake in humans (Degen et al. 2005) or rats (Stadlbauer et al. 2013) by an endocrine mechanism. In humans, anorexigenic doses of PYY(3‐36) produced systemic plasma levels of total PYY that were 2–3 times larger than those produced by a 1500 kcal meal (Degen et al. 2005). In rats, an anorexigenic dose of PYY(3‐36) produced systemic plasma levels of total PYY that were an order of magnitude larger than those produced by relatively small meals of either chow or high fat diet (Stadlbauer et al. 2013). Both studies used radioimmunoassays that measured total PYY in unextracted plasma. Our results here for total PYY extend these findings to show that plasma extraction enhanced the plasma PYY response to a large chow meal in rats, and that this response is likely to be an order of magnitude less than that produced by a minimal anorexigenic IV dose of PYY(3‐36). It is important to note that measures of total PYY in postprandial plasma by each of these assays may include similar amounts of active PYY(3‐36), inactive PYY(1‐36), and inactive metabolite PYY(3‐34), thus overestimating physiological levels of PYY(3‐36) (Toräng et al. 2015). Nevertheless, these results suggest that postprandial increases in PYY(3‐36) in systemic blood are not sufficient by themselves to inhibit food intake by an endocrine mechanism.

With respect to the pancreatic hormone amylin, we previously provided evidence that postprandial plasma levels of amylin are not quite sufficient to inhibit food intake in rats by an endocrine mechanism (Arnelo et al. 1998). Solid‐phase extraction of plasma and an amylin radioimmunoassay were used to show that a large chow meal increased plasma amylin from 43 to 62 pg/mL, while an anorexigenic IV dose of amylin (15 ng/kg/min) increased plasma amylin to 140 pg/mL. Here, we provide evidence using solid‐phase extraction of plasma and the multiplex assay for active amylin that a large chow meal increased plasma amylin from 34 to 140 pg/mL, and that a ~ED_50_ dose of amylin (39 ng/kg/min) produced 30‐fold higher levels. It remains to be determined whether IV infusion of amylin in a manner that mimics this postprandial increase, is sufficient to reduce food intake.

In summary, our results lead to the following conclusions: (1) Concentrations of peptide standards used in the individual ELIAs and multiplex assay differed significantly for GLP‐1, amylin, ghrelin, insulin, and GIP. (2) Solid‐phase extraction of lean and obese rat plasma samples to remove nonspecific interference prior to peptide assays produced consistent recoveries for GLP‐1, PYY, amylin, ghrelin, and insulin (but not for leptin and GIP), which reflect losses inherent to the extraction procedure. (3) Plasma extraction prior to peptide assays generally revealed larger meal‐induced changes in plasma levels of active GLP‐1, total PYY, active amylin, active ghrelin, and insulin in lean and DIO rats. (4) Plasma extraction and the multiplex assay were used to compare the plasma levels of active GLP‐1, total PYY, and active amylin after a large meal with plasma levels produced by IV infusions of anorexigenic doses of GLP‐1, PYY(3‐36), and amylin. Infusions produced dose‐dependent increases in plasma peptide levels, which were well above their postprandial levels. Together, these results do not support the hypothesis that postprandial plasma levels of GLP‐1, PYY(3‐36), and amylin are sufficient to decrease food intake by an endocrine mechanism.

## Conflict of Interest

The authors have no conflicts of interest to declare.

## References

[phy212800-bib-0001] Alsalim, W. , B. Omar , G. Pacini , R. Bizzotto , A. Mari , and B. Ahrén . 2015 Incretin and islet hormone responses to meals of increasing size in healthy subjects. J. Clin. Endocrinol. Metab. 100:561–568. 25375983 10.1210/jc.2014-2865

[phy212800-bib-0002] Amblard, M. , J. A. Fehrentz , J. Martinez , and G. Subra . 2006 Methods and protocols of modern solid phase Peptide synthesis. Mol. Biotechnol. 33:239–254. 16946453 10.1385/MB:33:3:239

[phy212800-bib-0003] Arnelo, U. , R. Reidelberger , T. E. Adrian , J. Larsson , and J. Permert . 1998 Sufficiency of postprandial plasma levels of islet amyloid polypeptide for suppression of feeding in rats. Am. J. Physiol. Regul. Integr. Comp. Physiol. 275:R1537–R1542. 10.1152/ajpregu.1998.275.5.R1537 9791071

[phy212800-bib-0004] Asmar, M. , M. Bache , F. K. Knop , S. Madsbad , and J. J. Holst . 2010 Do the actions of glucagon‐like peptide‐1 on gastric emptying, appetite, and food intake involve release of amylin in humans? J. Clin. Endocrinol. Metab. 95:2367–2375. 20194711 10.1210/jc.2009-2133

[phy212800-bib-0005] Bak, M. J. , N. J. Wewer Albrechtsen , J. Pedersen , F. K. Knop , T. Vilsbøll , N. B. Jørgensen , et al. 2014 Specificity and sensitivity of commercially available assays for glucagon‐like peptide‐1 (GLP‐1): implications for GLP‐1 measurements in clinical studies. Diabetes Obes. Metab. 16:1155–1164. 25041349 10.1111/dom.12352

[phy212800-bib-0006] Batterham, R. L. , M. A. Cohen , S. M. Ellis , C. W. Le Roux , D. J. Withers , G. S. Frost , et al. 2003 Inhibition of food intake in obese subjects by peptide YY3‐36. N. Engl. J. Med. 349:941–948. 12954742 10.1056/NEJMoa030204

[phy212800-bib-0007] Batterham, R. L. , H. Heffron , S. Kapoor , J. E. Chivers , K. Chandarana , H. Herzog , et al. 2006 Critical role for peptide YY in protein‐mediated satiation and body‐weight regulation. Cell Metab. 4:223–233. 16950139 10.1016/j.cmet.2006.08.001

[phy212800-bib-0008] Beglinger, C. , and L. Degen . 2006 Gastrointestinal satiety signals in humans–physiologic roles for GLP‐1 and PYY? Physiol. Behav. 89:460–464. 16828127 10.1016/j.physbeh.2006.05.048

[phy212800-bib-0009] Beglinger, S. , A. C. Meyer‐Gerspach , S. Graf , U. Zumsteg , J. Drewe , C. Beglinger , et al. 2014 Effect of a test meal on meal responses of satiation hormones and their association to insulin resistance in obese adolescents. Obesity (Silver Spring) 22:2047–2052. 24930697 10.1002/oby.20805

[phy212800-bib-0010] Brennan, I. M. , K. L. Feltrin , M. Horowitz , A. J. Smout , J. H. Meyer , J. Wishart , et al. 2005 Evaluation of interactions between CCK and GLP‐1 in their effects on appetite, energy intake, and antropyloroduodenal motility in healthy men. Am. J. Physiol. Regul. Integr. Comp. Physiol. 288:R1477–R1485. 15695321 10.1152/ajpregu.00732.2004

[phy212800-bib-0011] Chelikani, P. K. , A. C. Haver , and R. D. Reidelberger . 2005a Intravenous infusion of glucagon‐like peptide‐1 potently inhibits food intake, sham feeding, and gastric emptying in rats. Am. J. Physiol. Regul. Integr. Comp. Physiol. 288:R1695–R1706. 15718384 10.1152/ajpregu.00870.2004

[phy212800-bib-0012] Chelikani, P. K. , A. C. Haver , and R. D. Reidelberger . 2005b Intravenous infusion of peptide YY(3‐36) potently inhibits food intake in rats. Endocrinology 146:879–888. 15539554 10.1210/en.2004-1138

[phy212800-bib-0013] Cohen, J. 2003 Applied multiple regression. L. Erlbaum Associates, Mahwah, NJ.

[phy212800-bib-0014] Dai, H. , S. M. Gustavson , G. M. Preston , J. D. Eskra , R. Calle , and B. Hirshberg . 2008 Non‐linear increase in GLP‐1 levels in response to DPP‐IV inhibition in healthy adult subjects. Diabetes Obes. Metab. 10:506–513. 18284437 10.1111/j.1463-1326.2007.00742.x

[phy212800-bib-0015] Deacon, C. F. , and J. J. Holst . 2009 Immunoassays for the incretin hormones GIP and GLP‐1. Best Pract. Res. Clin. Endocrinol. Metab. 23:425–432. 19748060 10.1016/j.beem.2009.03.006

[phy212800-bib-0016] Degen, L. , S. Oesch , M. Casanova , S. Graf , S. Ketterer , J. Drewe , et al. 2005 Effect of peptide YY3‐36 on food intake in humans. Gastroenterology 129:1430–1436. 16285944 10.1053/j.gastro.2005.09.001

[phy212800-bib-0017] English, P. J. , A. Ashcroft , M. Patterson , T. M. Dovey , J. C. Halford , J. Harrison , et al. 2006 Fasting plasma peptide‐YY concentrations are elevated but do not rise postprandially in type 2 diabetes. Diabetologia 49:2219–2221. 16832662 10.1007/s00125-006-0344-y

[phy212800-bib-0018] Flint, A. , A. Raben , A. Astrup , and J. J. Holst . 1998 Glucagon‐like peptide 1 promotes satiety and suppresses energy intake in humans. J. Clin. Invest. 101:515–520. 9449682 10.1172/JCI990 PMC508592

[phy212800-bib-0019] Flint, A. , A. Raben , A. K. Ersbøll , J. J. Holst , and A. Astrup . 2001 The effect of physiological levels of glucagon‐like peptide‐1 on appetite, gastric emptying, energy and substrate metabolism in obesity. Int. J. Obes. Relat. Metab. Disord. 25:781–792. 11439290 10.1038/sj.ijo.0801627

[phy212800-bib-0020] Gibbons, C. , P. Caudwell , G. Finlayson , D. L. Webb , P. M. Hellström , E. Näslund , et al. 2013 Comparison of postprandial profiles of ghrelin, active GLP‐1, and total PYY to meals varying in fat and carbohydrate and their association with hunger and the phases of satiety. J. Clin. Endocrinol. Metab. 98:E847–E855. 23509106 10.1210/jc.2012-3835

[phy212800-bib-0021] Gutzwiller, J. P. , J. Drewe , B. Göke , H. Schmidt , B. Rohrer , J. Lareida , et al. 1999a Glucagon‐like peptide‐1 promotes satiety and reduces food intake in patients with diabetes mellitus type 2. Am. J. Physiol. Regul. Integr. Comp. Physiol. 276:R1541–R1544. 10.1152/ajpregu.1999.276.5.R1541 10233049

[phy212800-bib-0022] Gutzwiller, J. P. , B. Göke , J. Drewe , P. Hildebrand , S. Ketterer , D. Handschin , et al. 1999b Glucagon‐like peptide‐1: a potent regulator of food intake in humans. Gut 44:81–86. 9862830 10.1136/gut.44.1.81 PMC1760073

[phy212800-bib-0023] Halatchev, I. G. , and R. D. Cone . 2005 Peripheral administration of PYY(3‐36) produces conditioned taste aversion in mice. Cell Metab. 1:159–168. 16054059 10.1016/j.cmet.2005.02.003

[phy212800-bib-0024] Ito, T. , H. Thidarmyint , T. Murata , H. Inoue , R. M. Neyra , and H. Kuwayama . 2006 Effects of peripheral administration of PYY3‐36 on feed intake and plasma acyl‐ghrelin levels in pigs. J. Endocrinol. 191:113–119. 17065394 10.1677/joe.1.06855

[phy212800-bib-0025] Kinzig, K. P. , K. A. Scott , J. Hyun , S. Bi , and T. H. Moran . 2005 Altered hypothalamic signaling and responses to food deprivation in rats fed a low‐carbohydrate diet. Obes. Res. 13:1672–1682. 16286514 10.1038/oby.2005.205

[phy212800-bib-0026] Kuhre, R. E. , N. J. Wewer Albrechtsen , B. Hartmann , C. F. Deacon , and J. J. Holst . 2015 Measurement of the incretin hormones: glucagon‐like peptide‐1 and glucose‐dependent insulinotropic peptide. J. Diabetes Complications 29:445–450. 25623632 10.1016/j.jdiacomp.2014.12.006

[phy212800-bib-0027] Ludvik, B. , K. Thomaseth , J. J. Nolan , M. Clodi , R. Prager , and G. Pacini . 2003 Inverse relation between amylin and glucagon secretion in healthy and diabetic human subjects. Eur. J. Clin. Invest. 33:316–322. 12662162 10.1046/j.1365-2362.2003.01142.x

[phy212800-bib-0028] Meyer‐Gerspach, A. C. , B. Wölnerhanssen , B. Beglinger , F. Nessenius , M. Napitupulu , F. H. Schulte , et al. 2014 Gastric and intestinal satiation in obese and normal weight healthy people. Physiol. Behav. 129:265–271. 24582673 10.1016/j.physbeh.2014.02.043

[phy212800-bib-0029] Mietlicki‐Baase, E. G. , and M. R. Hayes . 2014 Amylin activates distributed CNS nuclei to control energy balance. Physiol. Behav. 136:39–46. 24480072 10.1016/j.physbeh.2014.01.013 PMC4113606

[phy212800-bib-0030] Näslund, E. , B. Barkeling , N. King , M. Gutniak , J. E. Blundell , J. J. Holst , et al. 1999 Energy intake and appetite are suppressed by glucagon‐like peptide‐1 (GLP‐1) in obese men. Int. J. Obes. Relat. Metab. Disord. 23:304–311. 10193877 10.1038/sj.ijo.0800818

[phy212800-bib-0031] Nauck, M. A. , U. Niedereichholz , R. Ettler , J. J. Holst , C. Orskov , R. Ritzel , et al. 1997 Glucagon‐like peptide 1 inhibition of gastric emptying outweighs its insulinotropic effects in healthy humans. Am. J. Physiol. Endocrinol. Metab. 273:E981–E988. 10.1152/ajpendo.1997.273.5.E981 9374685

[phy212800-bib-0032] Novelli, E. L. , Y. S. Diniz , C. M. Galhardi , G. M. Ebaid , H. G. Rodrigues , F. Mani , et al. 2007 Anthropometrical parameters and markers of obesity in rats. Lab. Anim. 41:111–119. 17234057 10.1258/002367707779399518

[phy212800-bib-0033] Østoft, S. H. , J. I. Bagger , T. Hansen , B. Hartmann , O. Pedersen , J. J. Holst , et al. 2015 Postprandial incretin and islet hormone responses and dipeptidyl‐peptidase 4 enzymatic activity in patients with maturity onset diabetes of the young. Eur. J. Endocrinol. 173:205–215. 25953829 10.1530/EJE-15-0070

[phy212800-bib-0034] Plamboeck, A. , S. Veedfald , C. F. Deacon , B. Hartmann , T. Vilsbøll , F. K. Knop , et al. 2015 The role of efferent cholinergic transmission for the insulinotropic and glucagonostatic effects of GLP‐1. Am. J. Physiol. Regul. Integr. Comp. Physiol. 309:R544–R551. 26136531 10.1152/ajpregu.00123.2015

[phy212800-bib-0035] Punjabi, M. , M. Arnold , E. Rüttimann , M. Graber , N. Geary , G. Pacheco‐López , et al. 2014 Circulating glucagon‐like peptide‐1 (GLP‐1) inhibits eating in male rats by acting in the hindbrain and without inducing avoidance. Endocrinology 155:1690–1699. 24601880 10.1210/en.2013-1447

[phy212800-bib-0036] Reidelberger, R. D. , U. Arnelo , L. Granqvist , and J. Permert . 2001 Comparative effects of amylin and cholecystokinin on food intake and gastric emptying in rats. Am. J. Physiol. Regul. Integr. Comp. Physiol. 280:R605–R611. 11171636 10.1152/ajpregu.2001.280.3.R605

[phy212800-bib-0037] Reidelberger, R. D. , A. C. Haver , U. Arnelo , D. D. Smith , C. S. Schaffert , and J. Permert . 2004 Amylin receptor blockade stimulates food intake in rats. Am. J. Physiol. Regul. Integr. Comp. Physiol. 287:R568–R574. 15130879 10.1152/ajpregu.00213.2004

[phy212800-bib-0038] Reidelberger, R. D. , A. C. Haver , B. A. Apenteng , K. L. Anders , and S. M. Steenson . 2011 Effects of exendin‐4 alone and with peptide YY(3‐36) on food intake and body weight in diet‐induced obese rats. Obesity (Silver Spring) 19:121–127. 20559304 10.1038/oby.2010.136

[phy212800-bib-0039] Reidelberger, R. , A. Haver , P. K. Chelikani , B. Apenteng , C. Perriotte‐Olson , K. Anders , et al. 2012 Effects of leptin replacement alone and with exendin‐4 on food intake and weight regain in weight‐reduced diet‐induced obese rats. Am. J. Physiol. Endocrinol. Metab. 302:E1576–E1585. 22510712 10.1152/ajpendo.00058.2012 PMC3378160

[phy212800-bib-0040] Reidelberger, R. , A. Haver , and P. K. Chelikani . 2013 Role of peptide YY(3‐36) in the satiety produced by gastric delivery of macronutrients in rats. Am. J. Physiol. Endocrinol. Metab. 304:E944–E950. 23482449 10.1152/ajpendo.00075.2013 PMC3651646

[phy212800-bib-0041] Reidelberger, R. , A. Haver , K. Anders , and B. Apenteng . 2014 Role of capsaicin‐sensitive peripheral sensory neurons in anorexic responses to intravenous infusions of cholecystokinin, peptide YY‐(3‐36), and glucagon‐like peptide‐1 in rats. Am. J. Physiol. Endocrinol. Metab. 307:E619–E629. 25117406 10.1152/ajpendo.00024.2014 PMC4200310

[phy212800-bib-0042] Rüttimann, E. B. , M. Arnold , J. J. Hillebrand , N. Geary , and W. Langhans . 2009 Intrameal hepatic portal and intraperitoneal infusions of glucagon‐like peptide‐1 reduce spontaneous meal size in the rat via different mechanisms. Endocrinology 150:1174–1181. 18948395 10.1210/en.2008-1221 PMC2654737

[phy212800-bib-0043] Shin, A. C. , H. Zheng , R. L. Townsend , D. L. Sigalet , and H. R. Berthoud . 2010 Meal‐induced hormone responses in a rat model of Roux‐en‐Y gastric bypass surgery. Endocrinology 151:1588–1597. 20179262 10.1210/en.2009-1332 PMC2850245

[phy212800-bib-0044] Stadlbauer, U. , M. Arnold , E. Weber , and W. Langhans . 2013 Possible mechanisms of circulating PYY‐induced satiation in male rats. Endocrinology 154:193–204. 23239815 10.1210/en.2012-1956

[phy212800-bib-0045] Talsania, T. , Y. Anini , S. Siu , D. J. Drucker , and P. L. Brubaker . 2005 Peripheral exendin‐4 and peptide YY(3‐36) synergistically reduce food intake through different mechanisms in mice. Endocrinology 146:3748–3756. 15932924 10.1210/en.2005-0473

[phy212800-bib-0046] Tolessa, T. , M. Gutniak , J. J. Holst , S. Efendic , and P. M. Hellström . 1998 Glucagon‐like peptide‐1 retards gastric emptying and small bowel transit in the rat: effect mediated through central or enteric nervous mechanisms. Dig. Dis. Sci. 43:2284–2290. 9790467 10.1023/a:1026678925120

[phy212800-bib-0047] Toräng, S. , S. Veedfald , M. M. Rosenkilde , B. Hartmann , and J. J. Holst . 2015 The anorexic hormone Peptide YY3‐36 is rapidly metabolized to inactive Peptide YY3‐34 in vivo. Physiol. Rep. 3:e12455. doi:10.14814/phy2.12455. 26197931 10.14814/phy2.12455 PMC4552532

[phy212800-bib-0048] Verdich, C. , S. Toubro , B. Buemann , J. Lysgård Madsen , J. Juul Holst , and A. Astrup . 2001 The role of postprandial releases of insulin and incretin hormones in meal‐induced satiety–effect of obesity and weight reduction. Int. J. Obes. Relat. Metab. Disord. 25:1206–1214. 11477506 10.1038/sj.ijo.0801655

[phy212800-bib-0049] Vilsbøll, T. , T. Krarup , C. F. Deacon , S. Madsbad , and J. J. Holst . 2001 Reduced postprandial concentrations of intact biologically active glucagon‐like peptide 1 in type 2 diabetic patients. Diabetes 50:609–613. 11246881 10.2337/diabetes.50.3.609

[phy212800-bib-0050] Vilsbøll, T. , T. Krarup , J. Sonne , S. Madsbad , A. Vølund , A. G. Juul , et al. 2003 Incretin secretion in relation to meal size and body weight in healthy subjects and people with type 1 and type 2 diabetes mellitus. J. Clin. Endocrinol. Metab. 88:2706–2713. 12788877 10.1210/jc.2002-021873

[phy212800-bib-0051] Williams, D. L. , D. G. Baskin , and M. W. Schwartz . 2009 Evidence that intestinal glucagon‐like peptide‐1 plays a physiological role in satiety. Endocrinology 150:1680–1687. 19074583 10.1210/en.2008-1045 PMC2659282

[phy212800-bib-0052] Witte, A. B. , P. Grybäck , J. J. Holst , L. Hilsted , P. M. Hellström , H. Jacobsson , et al. 2009 Differential effect of PYY1‐36 and PYY3‐36 on gastric emptying in man. Regul. Pept. 158:57–62. 19651163 10.1016/j.regpep.2009.07.013

[phy212800-bib-0053] Woltman, T. , D. Castellanos , and R. Reidelberger . 1995 Role of cholecystokinin in the anorexia produced by duodenal delivery of oleic acid in rats. Am. J. Physiol. Regul. Integr. Comp. Physiol. 269:R1420–R1433. 10.1152/ajpregu.1995.269.6.R1420 8594945

[phy212800-bib-0054] Yabe, D. , A. Kuroe , S. Lee , K. Watanabe , T. Hyo , M. Hishizawa , et al. 2010 Little enhancement of meal‐induced glucagon‐like peptide 1 secretion in Japanese: comparison of type 2 diabetes patients and healthy controls. J. Diabetes Investig. 1:56–59. 10.1111/j.2040-1124.2010.00010.x PMC4020678 24843409

[phy212800-bib-0055] Yabe, D. , K. Watanabe , K. Sugawara , H. Kuwata , Y. Kitamoto , K. Sugizaki , et al. 2012 Comparison of incretin immunoassays with or without plasma extraction: incretin secretion in Japanese patients with type 2 diabetes. J. Diabetes Investig. 3:70–79. 10.1111/j.2040-1124.2011.00141.x PMC4014935 24843548

[phy212800-bib-0056] Yabe, D. , A. Kuroe , K. Watanabe , M. Iwasaki , A. Hamasaki , Y. Hamamoto , et al. 2015 Early phase glucagon and insulin secretory abnormalities, but not incretin secretion, are similarly responsible for hyperglycemia after ingestion of nutrients. J. Diabetes Complications 29:413–421. 25613093 10.1016/j.jdiacomp.2014.12.010

[phy212800-bib-0057] Young, A. 2005 Tissue expression and secretion of amylin. Adv. Pharmacol. 52:19–45. 16492539 10.1016/S1054-3589(05)52002-7

[phy212800-bib-0058] Zhang, J. , and R. C. Ritter . 2012 Circulating GLP‐1 and CCK‐8 reduce food intake by capsaicin‐insensitive, nonvagal mechanisms. Am. J. Physiol. Regul. Integr. Comp. Physiol. 302:R264–R273. 22031786 10.1152/ajpregu.00114.2011 PMC3349390

